# Hodgkin lymphoma and liquid biopsy: a story to be told

**DOI:** 10.1186/s13046-024-03108-6

**Published:** 2024-07-02

**Authors:** Jesús Velasco-Suelto, Laura Gálvez-Carvajal, Iñaki Comino-Méndez, Antonio Rueda-Domínguez

**Affiliations:** 1 Unidad de Gestion Clinica Intercentros de Oncologia Medica, Hospitales Universitarios Regional y Virgen de La Victoria, 29010, Malaga, Spain; 2 The Biomedical Research Institute of Málaga, IBIMA-CIMES-UMA), 29010 Malaga, Spain; 3 Andalusia-Roche Network in Precision Medical Oncology, 41092 Seville, Spain; 4 Centro de Investigacion Biomedica en Red de Cancer (CIBERONC - CB16, 12/00481); 28029, Madrid, Spain; 5 Clinical and Translational Cancer Research Group, IBIMA Institute, C/ Severo Ochoa, ParqueTecnologico de Andalucia (PTA), 35, 29590 Campanillas-Malaga, Spain

**Keywords:** Liquid biopsy, Hodgkin lymphoma, Circulating free DNA, Circulating tumor DNA, PET/CT, miRNAs, cytokines

## Abstract

Hodgkin lymphoma (HL) represents a neoplasm primarily affecting adolescents and young adults, necessitating the development of precise diagnostic and monitoring tools. Specifically, classical Hodgkin lymphoma (cHL), comprising 90% of cases, necessitating tailored treatments to minimize late toxicities. Although positron emission tomography/computed tomography (PET/CT) has enhanced response assessment, its limitations underscore the urgency for more reliable progression predictive tools. Genomic characterisation of rare Hodgkin Reed-Sternberg (HRS) cells is challenging but essential. Recent studies employ single-cell molecular analyses, mass cytometry, and Next-Generation Sequencing (NGS) to unveil mutational landscapes. The integration of liquid biopsies, particularly circulating tumor DNA (ctDNA), extracellular vesicles (EVs), miRNAs and cytokines, emerge as groundbreaking approaches. Recent studies demonstrate ctDNA's potential in assessing therapy responses and predicting relapses in HL. Despite cHL-specific ctDNA applications being relatively unexplored, studies emphasize its value in monitoring treatment outcomes. Overall, this review underscores the imperative role of liquid biopsies in advancing HL diagnosis and monitoring.

## Background

### Hodgkin lymphoma in clinic

Hodgkin lymphoma (HL) is a neoplasm with an annual incidence of three cases per 100,000 individuals, primarily affecting adolescents and young adults in Western society [1, 2]. There are two principal types of Hodgkin lymphoma: nodular lymphocyte-predominant Hodgkin lymphoma (NLPHL) and classical Hodgkin lymphoma (cHL). On one hand, 5–10% of cases are NLPHL, characterised by the proliferation of small lymphocytes with scattered large neoplastic cells known as lymphocyte-predominant (LP) cells [1]. On the other hand, more than 90% of cases are cHL, which is divided into four subtypes based on their morphology and immunohistochemistry: nodular sclerosis cHL (NSCHL), mixed cellularity cHL (MCCHL), lymphocyte-depleted cHL (LDCHL), and lymphocyte-rich cHL (LRCHL) [1, 3, 4]. While it is not always possible to distinguish between these subtypes, it is essential to differentiate cHL from NLPHL [3].

cHL is characterised by the presence of markedly rare malignant cells known as Hodgkin and Reed-Sternberg (HRS) cells. These cells have a B lymphocyte origin and are characterised by an increase in size and number of nuclei. The HRS cell microenvironment is composed of a variety of non-malignant immune effector cells such as T cells, B cells, eosinophils, macrophages, and fibroblasts [4–6]. In tissues, HRS cells constitute approximately 1% of the tumor complicating their molecular characterisation due to their scarcity [1, 4, 7]. Specifically, HRS cells form rosettes and interact with surrounding lymphocytes [8]. The origin of this disease has no definitive evidence, but it is believed to result from a combination of genetic, environmental, and immune system factors. It has also been suggested that in some cases, the Epstein-Barr Virus (EBV) [4, 7, 9] or human immunodeficiency virus [10] could be involved.

The initial treatment for cHL typically involves chemotherapy, with or without radiotherapy; however, other treatments such as monoclonal antibodies (BV) and immunotherapy could be also indicated. This therapy has proven effective, achieving remission in over 90% of cases and curing at least 80% of patients when considering all disease stages[11]. However, the treatment's effectiveness and aggressiveness, combined with poorly understood factors[12], mean that cHL is directly responsible for less than half of patient deaths[13]. This is particularly notable in patients with early-stage disease, where lymphoma progression accounts for less than one-third of fatalities[14].

The primary cause of death in cHL is the late toxic effects of treatment [15], which typically manifest after the fifth year of follow-up and steadily increase, even beyond 30 years from the initial treatment. Common late effects include secondary malignancies (such as solid tumors and acute leukaemia) and severe cardiovascular events (such as ischemic heart disease, stroke, and heart failure), primarily induced by the treatment, particularly radiotherapy [16].

Since the late twentieth century, a key focus of clinical research in cHL has been tailoring each patient's treatment based on their risk of lymphoma-related death. This approach aims to provide more aggressive treatments to patients with a poorer prognosis and less intensive treatments to those with a more favourable outlook, with the goal of reducing severe late side effects [17].

Since 2007, PET/CT (positron emission tomography/computed tomography) has emerged as the preferred tool for assessing the response in classical Hodgkin lymphoma (cHL). Interim PET/CT (iPET, PET performed after the first two cycles of chemotherapy) has gained acceptance as a valuable tool for adjusting treatment intensity based on the observed response [18].

iPET exhibits a robust negative predictive value (NPV) when evaluating the response of cHL, allowing for treatment de-escalation while maintaining a high cure rate [19, 20]. However, the positive predictive value (PPV) of iPET falls short of clinical utility, primarily due to its limited accuracy (approximately 40–50% after ABVD treatment)[21–23], resulting in relapse rates ranging from 15 to 60% [20, 22, 23].

Approximately 50% of patients with positive iPET results will remain free from progression, while 20–25% of patients with negative iPET will experience disease progression [24]. Given the substantial rate of false positives and the association between more intensive treatment regimens and both improved cure rates and heightened acute and long-term toxicity, there is an imperative need for a more dependable predictive tool for disease progression.

While FDG-PET (18-Fluoro-deoxyglucose positron emission tomography) is recognized as a valuable tool for detecting and monitoring responses to treatment in cHL and other lymphomas, some authors argue that it does not offer significant advantages over physical examination-based methods for detecting relapses [25–27]. Nonetheless, this procedure raises several issues for patients, including radiation exposure and the potential for false-positive results [28]. Importantly, this approach may not reliably detect relapses and may increase the risk of second malignancies, especially in pediatric and young adult patients. To underscore the radiation exposure concern, Pingali et al. determined that the cumulative exposure from additional scans is equivalent to 27 years of background radiation [25]. Furthermore, considering the issue of false-positives, two studies found a high proportion of patients with these erroneous scan results, often due to infectious and inflammatory processes, leading to unnecessary treatment changes for patients who were actually free of disease [29, 30].

**Fig. 1:**
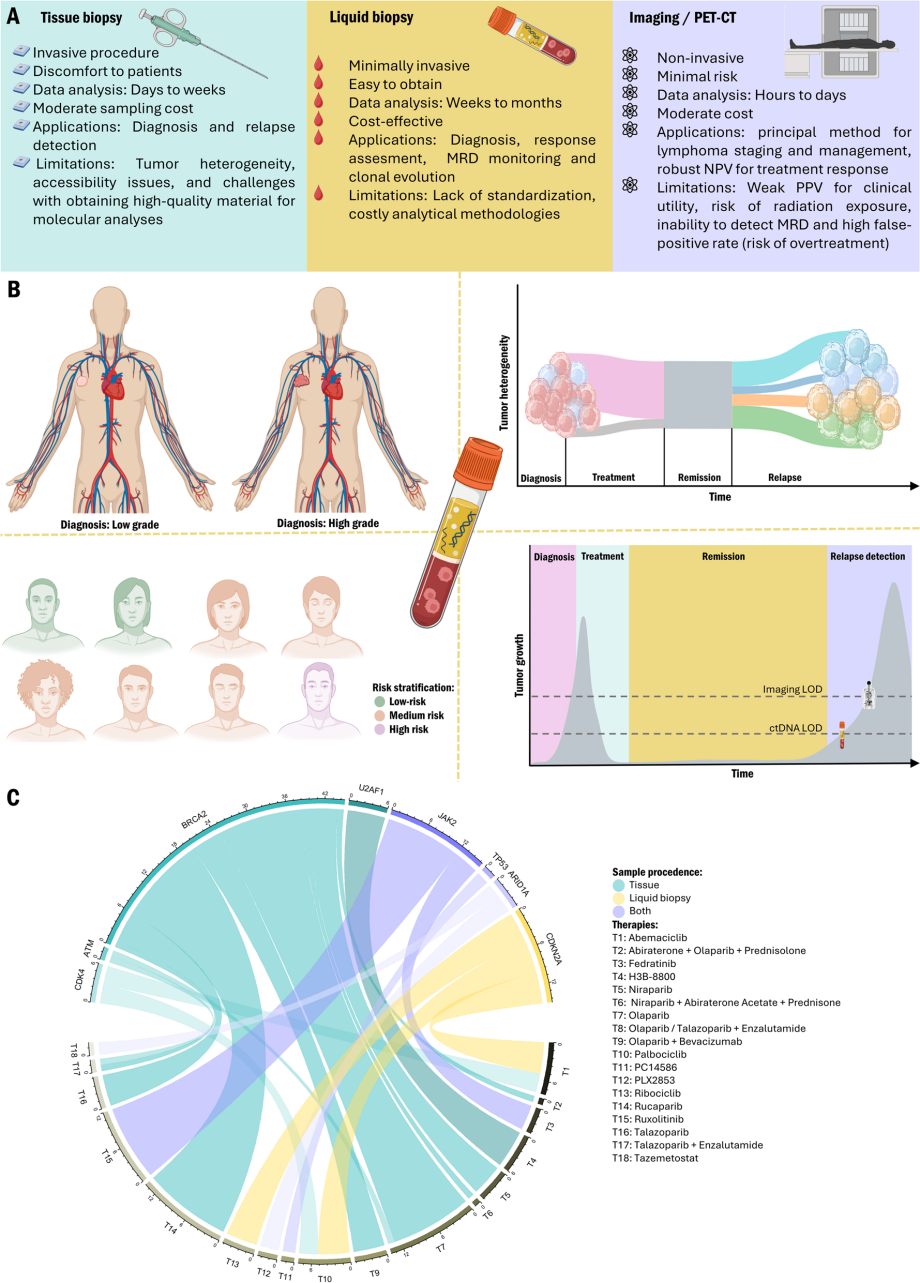
Potential applications and limitations of different methodologies for the therapeutic management of Hodgkin lymphoma. **A** Comparison of tumor biopsy, liquid biopsy, and Imaging/PET-CT. **B** Potential uses of liquid biopsy to diagnose the disease, stratify patients, decipher tumor heterogeneity, monitor disease response, and predict relapse. **C** Circos plot illustrating the genes with mutations found in solid biopsy (teal), circulating free DNA (yellow), and both (purple) that are linked to any therapy. The recommended therapies for each mutated gene are depicted from T1 to T18. For the genes, the width of the lines corresponds to the total number of citations linking the gene with a given therapy. Conversely, the width of the lines for the treatments corresponds to the number of citations for each therapy (https://www.oncokb.org/). *LOD: Limit of detection; MRD: Minimal Residual Disease; NPV: Negative predictive value; PPV: Positive predictive value*

In light of these considerations, there is an urgent and pressing need to develop innovative and non-invasive tools that can sensitively monitor the presence and progression of the disease during and after treatments (Fig. 1).

### Molecular profile of HRS cells and tumor microenvironment

The diagnosis of cHL is made by a tissue biopsy as it is essential to identify the presence of HRS cells. Not only the diagnosis based on HRS but also the tumor microenvironment (TME) characterization is essential to differentiate between subtypes. The TME contributes to refractoriness to therapy, relapse or even poor survival rates in cHL [31]. Considering the above-mentioned caveat on tumor cells rarity in the tissue, the molecular characterization of HRS cells and TME, it is essential to identify valuable targets for precision therapy of patients with cHL. Some of the latest studies are based on single-cell molecular analysis, employing technologies such as mass cytometry (CyTOF), single-cell RNA sequencing (scRNA-seq), and multiplexed imaging. They also permit the study of the TME, paving the way for a better understanding of this disease [32]. However, there is currently no evidence of using the molecular characterization of these cells to implement and improve the clinical management of patients with cHL.

In this context, Next-Generation Sequencing (NGS) is a powerful tool, not only for diagnosis but also for monitoring treatment responses and stratifying patients based on their risk of relapse [33]. NGS has begun to shed light on the dynamic nature of this disease and has been used to characterize the mutational landscape of HRS cells. For instance, in a study conducted by Reichel et al., HRS cells were sequenced, revealing alterations in genes related to the immune system, genomic stability, and transcriptional regulation. The authors identified that inactivating mutations in the *B2M* gene resulted in major histocompatibility complex I (MHC-I) downregulation. Notably, they found that *B2M* inactivating mutations and alterations in the NF-κB pathway were more prevalent in the nodular sclerosis cHL (NSCHL) subtype, whereas certain cases classified as mixed cellularity cHL (MCCHL) did not exhibit a characteristic mutational landscape, suggesting molecular heterogeneity within this subtype [33].

In another study, a whole-exome sequencing (WES) analysis of HRS cells isolated by microdissection identified increased copy number alterations (CNAs) affecting the *JAK2* gene, an enrichment of mutations in the *STAT6* gene, and activating mutations in genes such as *JAK1* and other transcription factors from the JAK-STAT signaling pathway, including *STAT3* and *STAT5B*, in approximately 90% of cases. In this study, the most prevalent mutations were found in the *GNA13* (24%), *XPO1* (18%), *ITPKB* (16%), and *STAT6* (32%) genes [34]. Additionally, Wienand et al. conducted WES on HRS cells isolated by flow cytometry. The authors observed frequent mutations in the *B2M* (39%), *NFKBIE* (26%), *TNFAIP3* (26%), and *NFKBIA* (17%) genes. Consistent with previous reports, they also identified mutations in the JAK/STAT pathway, including mutations in the *SOCS1* (70%) and *STAT6* (35%) genes. Furthermore, somatic CNAs were observed, with frequencies ranging from 9 to 52%. One of the most frequent CNAs involved arm-level 9 gain and focal amplification of the 9p24.1/PD-L1/PD-L2/JAK2 region [35].

Collectively, these studies have illuminated the intricate genomic landscape of HRS cells. In this context, Mangano et al. introduced an innovative methodology utilizing single-cell isolation of these rare and scarce HRS cells through the use of DEPArray™, an image-based cell-sorting technology. This approach allowed for the isolation of individual cells from cHL tissues, enabling the study of their CNAs. Their investigation revealed common altered regions on chromosomes 2p, 8q, and 9q. Notably, these regions encompass genes known to be frequently altered in cHL, including those associated with the REL/ NF-κB and JAK/STAT pathways [36]. Elevated levels of genetic imbalances were also identified in oncogenes previously recognized as being altered in cHL, including *MDM4* and *U2AF1*. This serves as an illustrative example of how innovative, high-precision single-cell isolation technologies can contribute to the comprehensive characterization of HRS cells. Such advancements not only enhance our understanding of cHL but also pave the way for the development of personalized therapeutic and disease-monitoring strategies (Fig. 1).

The challenges in characterizing HRS cells and translating this knowledge into clinical practice for the benefit of patient management are evident. In some recent significant study, investigators have demonstrated the feasibility of characterizing this disease through liquid biopsy, analyzing circulating tumor DNA to monitor therapy responses, establishing risk-of-relapse stratification and detecting minimal residual disease. Further discussion of this article will follow in the subsequent Sect. [37].

### Liquid biopsy in lymphomas as an emerging diagnostic and monitoring tool

Liquid biopsy has emerged as a promising diagnostic and monitoring approach for the detection and characterization of cancers using bodily biofluids, such as blood. In the context of solid tumors, this methodology enables a more comprehensive characterization of the tumor’s genetic heterogeneity [38]. Furthermore, it offers the advantage of improved accessibility for serial monitoring of cancer progression (Fig. 1).

The translation of liquid biopsy into the concept of precision medicine and routine clinical practice holds significant potential, particularly in the context of detecting minimal residual disease (MRD) with the goal of identifying relapses before they manifest clinically [38, 39]. Specifically, the analysis of circulating tumor DNA (ctDNA) has emerged as a promising tool for cancer characterization, patient stratification, and the early detection of disease relapse [40–42] (Fig. 1).

In lymphoma research, a notable study has showcased the utility of ctDNA detection as an effective tool for disease monitoring in diffuse large B-cell lymphoma (DLBCL). This study provided compelling evidence that ctDNA during treatment and surveillance is a useful biomarker to predict therapy failure and risk of future relapse before clinically evident exhibiting enhanced sensitivity when compared to FDG-PET imaging [43]. In this context, one of the most recent research endeavors was conducted by Jimenez-Ubieto et al. Their study involved sequencing patients with follicular lymphoma (FL) to identify somatic mutations that could serve as personalized MRD assays in plasma. Following the design of a specific panel, they conducted sequencing at an ultra-high depth, achieving a remarkable sensitivity of 2 × 10^–4^. The results were notable; their MRD assay, when coupled with PET/CT imaging analysis, effectively detected patients who experienced relapses in less than two years with an impressive sensitivity of 88% and a specificity of 100%. This serves as a compelling example of how liquid biopsy, personalized NGS assays for ctDNA detection, and current imaging tools can collectively enable the stratification of lymphoma patients based on their risk of relapse [44].

In case of cHL, there are no experimental evidence of using advanced personalized NGS assays to monitor disease response and relapse monitoring. However, some studies have described the mutational landscape of ctDNA using NGS in this patient’s type. Certain mutations affecting genes such as *SOCS1*, *STAT6*, *XPO1*, *TNFAIP3*, *NFKBIE*, *B2M*, *NOTCH* and *PI3K* were observed in plasma from patients with cHL [45–50]. Indeed, it has been shown that ctDNA levels are correlated with metabolic tumor volumes and disease outcomes [46]. In this regard, Alcoceba et al., employed a targeted capture NGS panel for liquid biopsy including coding regions and splice sites of 37 genes, and hot-spots for an additional five genes involved in cHL. In detail, they detected ctDNA in 73.5% of samples at diagnosis and five variants per case with VAFs ranging from 0.84% to 28%. They observed mutations in most of the previously mentioned genes but also an association of higher ctDNA levels with poor prognosis clinical signatures [51].

In a groundbreaking study, Spina et al. conducted an in-depth investigation of ctDNA cHL through comprehensive sequencing. Their research aimed to establish the correlation between mutations found in tumor tissue and those in ctDNA, decipher the mutational landscape of ctDNA, and assess the potential of ctDNA for prognostic purposes in cHL. To achieve this, they utilized a non-specific targeted panel designed for mature B-cell tumors, complemented by ultra-deep sequencing techniques. The study revealed a robust correlation between genetic aberrations present in HRS cells and those found in ctDNA. Interestingly, their findings identified *STAT6* as the most frequently altered gene in cHL, challenging previous research findings. This study proposes the measurement of ctDNA as a radiation-free tool for tracking residual disease, offering significant potential in cHL prognosis [52]. In detail, they observed a 100-fold drop in ctDNA as a marker to predict progression in their cohort that was also associated with complete response and cure. This observation was as also reported in previous investigations in other lymphoma types [53].

In another important study, Shi et al., employed a specific fixed target panel, including common genes affected in cHL and other common lymphomas and hematologic malignancies. Herein, they characterized the mutational landscape of cHL by ctDNA sequencing but also evaluated the capacity of ctDNA to predict immunotherapy treatment response and disease recurrence. In detail, they observed that: i) mutations affecting the gene *CHD8* were significantly associated with longer progression-free survival (PFS), ii) baseline ctDNA was significantly higher in responders to therapy and iii) a decrease in ctDNA levels of ≥ 40% from baseline indicated better outcome. Furthermore, they propose that mutations in the *B2M*, *TNFRSF14* and *KDM2B* genes are associated with acquired resistance to this particular treatment [47].

In another study conducted by Buedts et al., they analyzed CNAs in cell-free DNA (cfDNA) from cHL patients compared to healthy individuals. Approximately 90% of patients presented CNAs in cfDNA, with the detection of new recurrent CNAs such as gain of 15q21-q26 and the loss of 3p13-p26 and 12q21-q24. Interestingly, they discovered that CNAs and ctDNA levels decrease after treatment initiation, being present only in those patients with a higher probability of relapse [54]. Within the context of CNAs study in cfDNA, Raman et al. carried out a shallow-depth sequencing for tumor heterogeneity characterization, demonstrating that liquid biopsy-derived CNAs could differentiate between HL and DLBCL cases. Additionally, the results of the analysis of longitudinal samples suggest that CNAs patterns were similar across patients who were more likely to experience a relapse [55].

In a recent study, among other aspects, researchers investigated the clearance of ctDNA in previously untreated patients with cHL treated with the novel therapy involving pembrolizumab and chemotherapy. The study findings revealed that ctDNA clearance, observed after cycle two and at the end of treatment, was significantly associated with superior PFS. Furthermore, it's noteworthy that patients who exhibited imaging positivity but tested ctDNA-negative in plasma did not experience relapses by the time of the article's publication [56].

Finally, a recent investigation has explored the challenges associated with comprehensive genomic profiling of cHL through ctDNA characterization instead of tumor tissue. This study underscores the potential of liquid biopsies for molecular profiling of cHL. On one hand, the investigation revealed, through single-cell transcriptional profiles, that high ctDNA shedding in this tumor type is influenced by DNASE1L3 expression. Secondly, analysis of plasma samples from 366 patients identified two distinct genomic subtypes of cHL with clinical and prognostic implications, alongside novel *IL4R* mutations potentially targetable with IL-4Rα-blocking antibodies. The study also showcases the clinical value of pretreatment and on-treatment ctDNA levels for refining risk prediction and detecting minimal residual disease using an ultrasensitive technology called PhasED-seq. In this context, the researchers concluded that ctDNA levels have the potential to refine staging procedures, complement current risk stratification tools like iPET, and guide the selection of appropriate therapies [37].

Other studies investigated the association of cfDNA/ctDNA with other biological or imaging tools involved in cHL. In the field of pediatric cHL, the amount of cfDNA and ctDNA in patients with cHL and healthy individuals was investigated. It was shown that the cfDNA levels were higher in patients with cHL but also were correlated with poor prognosis [57, 58]. In an additional study, the authors evaluated the capacity of EBV and/or cfDNA detection and quantification in blood to provide insights into prognosis and treatment response. In concordance with the previous studies, cfDNA was elevated in cHL compared to controls [59]. Recently, there has been an exploration of the associations between PET/CT parameters and ctDNA in HL. Studies have delved into parameters related to tumor burden, tumor location, and dispersion in conjunction with ctDNA measurements. The findings suggest that quantifying ctDNA could provide additional value to traditional PET/CT, leading to better stratification and enhanced clinical management for patients with cHL [60]. In another innovative study including iPET analysis involving patients with relapsed/refractory cHL, the authors focused on identifying reliable biomarkers for treatment failure in relapsed/refractory cHL. Analyzing 55 patients treated with the bendamustine, gemcitabine and vinorelbine (BEGEV) regimen, researchers found that baseline ctDNA genotyping mirrored gene mutations in newly diagnosed cHL. Baseline ctDNA quantification and serial monitoring proved prognostic in these patients undergoing salvage chemotherapy. Integrating ctDNA with iPET enhanced early identification of high-risk patients, suggesting potential benefits from an early switch to immunotherapeutic agents [61].

Currently, there are several significant studies assessing the potential of plasma DNA as a valuable tool for therapy response assessment and relapse monitoring. Nevertheless, a majority of these studies have employed non-specific approaches or utilized short gene panels, particularly in the context of cHL. In light of this, high-throughput methodologies, such as whole-genome sequencing (WGS), which also encompasses the analysis of CNAs, could greatly contribute to unraveling the complex genomic landscape of cHL. This, in turn, may facilitate the design of highly personalized gene panels for use in ctDNA monitoring. However, the detection and on-treatment monitoring of ctDNA demand the expertise of highly skilled personnel, cutting-edge technologies, and proficiency in bioinformatics. In contrast, the quantification of total cfDNA involves a more straightforward methodology, commonly utilizing fluorometric assays or PCR-based platforms for measurement. As previously mentioned, various studies have investigated total cfDNA levels in patients with cHL, particularly at pre-treatment stages, revealing associations with advanced disease stages, unfavorable outcomes, and treatment failures, among other factors [54, 57, 58]. Some investigations have delved into the correlation between fluctuations in total cfDNA levels during therapy and subsequent treatment responses [58, 59]. It is crucial to emphasize that further extensive studies, encompassing larger cohorts, are imperative to validate these findings. Consequently, the comprehensive measurement of cfDNA presents itself as a pragmatic and readily implementable approach in clinical settings over the short term.

It is worth noting that liquid biopsy, specifically the detection and characterization of ctDNA, has demonstrated substantial promise in elucidating the underlying genomic mechanisms of tumor pathology. It holds significant potential for aiding in the monitoring of treatment responses and the early detection of relapses. However, implementing liquid biopsy and ctDNA in clinical practice involves addressing significant challenges in the preanalytical, analytical, and postanalytical phases. In the preanalytical phase, standardized protocols for sample selection, handling, processing, and storage are crucial to minimize errors. Validating these protocols is essential for optimal mutation detection. Biological variability in biofluids poses additional challenges, and choosing the appropriate biofluid for biomarker discovery requires considering factors like tumor location and accessibility. Standardization in the preanalytical phase is key for ensuring the reproducibility and reliability of liquid biopsy studies [62].

In the analytical phase, critical factors must be addressed, including quantification and qualification of cfDNA. Methods such as fluorescence-based assays and PCR-based tools are vital for assessing sample suitability. The choice of ctDNA analysis method, whether tumor-informed or tumor-agnostic, adds complexity to homogenization efforts. Additionally, reference materials and the measure of analytical outcome, particularly in terms of quantitative potential and result presentation, further highlight the challenges in standardizing liquid biopsy procedures [63].

In the postanalytical phase, factors crucially affect sensitivity and specificity. Processes like fragmentomic and bioinformatic pipelines enhance ctDNA detection accuracy. Meticulous evaluation of detected variants, including addressing artifacts and applying in silico size-selection, is essential. In addition, comprehensive diagnostic molecular reports, are necessary for accurate interpretation. Furthermore, standardization remains a significant challenge, requiring the development and validation of protocols through interlaboratory studies. In this regard, collaborative efforts involving international consortia are imperative for establishing universally applicable guidelines for clinical implementation [63].

**Fig. 2:**
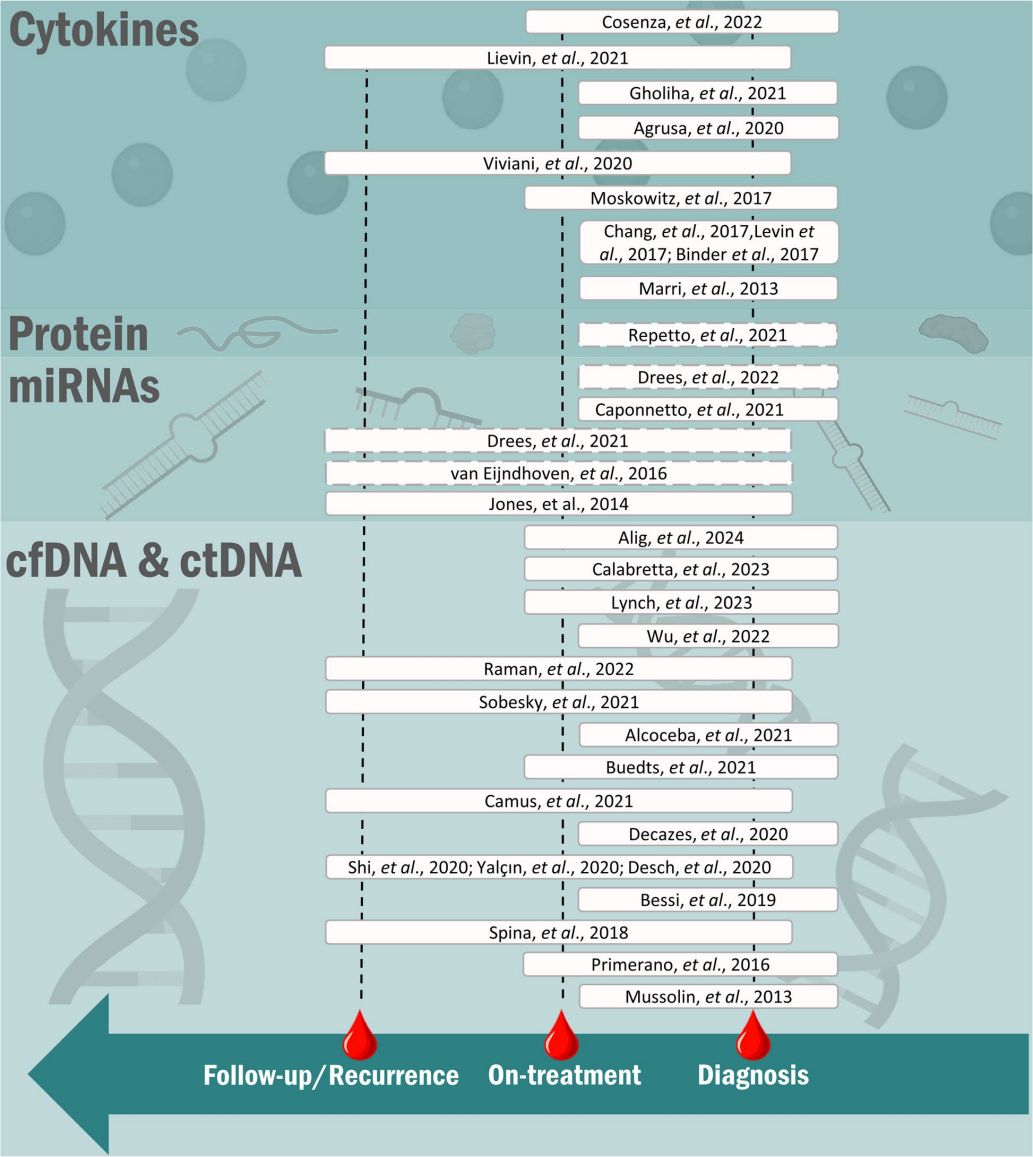
Diagram illustrating the primary advantages of liquid biopsy in oncology and the main published studies. Previous selected investigations that utilized cytokines, proteins, miRNAs, and circulating DNAs are depicted along with a reference to the specific timepoint during the patient's clinical course when the blood samples were extracted and studied. Studies in dashed squares explore the role of extracellular vesicles in HL

The previously mentioned studies are summarized in Table 1 and depicted in Fig. 2.

**Table 1:** Liquid biopsy in lymphomas as an emerging diagnostic and monitoring tool

Reference	Method/s employed	Observed detection values: LOD, sensitivity, specificity, etc	Samples	Altered genes/genomic regions	Main findings
[37]	- Capture targeting panels: EBV, SCNA, TCR sequencing, SABER - EPIC-seq and scRNAseq - PhasED-seq	RePhyNER: Sensitivity: 91% Specificity: 99% SNVs in plasma: VAF ≥ 0.5% Phased-variants: VAF ≥ 0.5% Genotyping and non-silent mutations: Minimum VAF: 0,5% EBV positivity threshold: 1.5 log_10_ EBV reads per ml plasma	Plasma samples: - Diagnosis (*n* = 366) - On-treatment (*n* = 310 samples, 109 patients)	- Mutations: *SOCS1* (60%), *TNFAIP3* (50%), *B2M* (39%), *STAT6* (34%), *CSF2RB* (24%), *GNA13* (23%), *PTPN1* (18%), *ARID1A* (17%), *ZNF217* (14%), *IL4R* (10%), *NFKBIA* (9%), *ACTB* (9%), *PCBP1* (8%), *CISH* (6%), *NFKB2* (6%), linker histone *H1-5* (6%) and *CD74* (3%) - Amplifications: 2p15 (*REL*), 9p24.1–9p24.2 (*PDL1*), 5p15.33 (*TERT*) and 17q21.31 (*MAP3K14*) - Deletions: 6q27 (*TNFAIP3*), 17p13.1 (*TP53*), 9p21.3 (*CDKN2A/B*), 11q22.3 (*BIRC3*) and 6p21–22 (*H1-5*, *HLA-A* and *HLA-C)*	- cHL blood samples present an enrichment in mutations compared with corresponding bulk tumors - For shared mutations between tissue and plasma samples, plasma VAFs exceed tumor VAFs in 79% of cases, suggesting a better genotyping approach over bulk tissue-based methods - DNASE1L3 could be related to ctDNA increase and maintaining the supportive tumor microenvironment - Identification of two distinct cHL genetic subtypes (H1 and H2) - H1 is driven by NFkB signaling, crosstalk signaling within the cHL tumor microenvironment, cytokine receptors and downstream STAT signaling - H2 subtype presents more conventional lymphoma drivers such us *KMT2D* and *TP53* - Discovery of a novel class of truncating a recurrent cHL variant defined by ctDNA, IL-13 cytokine-dependant *IL4R* truncating mutations - Prognostic potential of pretreatment and on-treatment ctDNA levels analysis in cHL management
[45]	NGS panel including regions of *B2M*, *STAT6*, *XPO1*, *NFKBIE*, *PTPN1* and *TNFAIP3* genes and ddPCR targeting the mutation N417Y affecting the *STAT6* gene	NGS LOD tissue VAF: ≥ 0.06% NGS LOD cfDNA mean VAF: 0.363% Mean cfDNA: 36.2 ng/mL (range, 17.36–61.2)	Plasma samples: - Diagnosis (*n* = 24)	*- STAT6*: 12 mutations (37.5%) *- XPO1*: (37.5%), six cases with E571K mutation (25%) *- B2M*: (29.2%) - GTAA deletion on exon 1 affecting *NFKBIE* (8.3%) *- PTPN1* (29.2%) *- TNFAIP3* (29.2%)	- Plasma as a source of tumor DNA for cHL genotyping - Reports the detection of mutations in both tissue biopsy DNA and cfDNA and the challenges in achieving sufficient sensitivity - Provides details about the types and patterns of mutations detected, including those in *STAT6*, *XPO1*, *B2M*, and more - Describes the use of ddPCR for monitoring MRD in cHL patients with recurrent mutations
[46]	HC-tNGS including genes recurrently mutated in B-cell lymphoma and genomic breakpoints of translocation and fragments of clonotypic Ig-gene rearrangements	Sensitivity (VAF): ≥ 0.5%	Plasma samples: - Diagnosis (*n* = 96) - On-treatment (*n* = 122) - Follow-up/Recurrence (*n* = 30)	- *SOCS1* (83%) - *STAT6,* two variants on the same allele in 7 out of 19 cases - *XPO1* - Predominantly inactivating mutations: *SOCS1*, *TNFAIP3*, *NFKBIE*, *B2M*, *NFKBIA*, *ARID1A* and *PTPN1*	- Detected HRS cell-derived SNVs, indels, translocations, and *VH-DH-JH* rearrangements in pretherapy cfDNA of a significant number of patients - Found a range of variants per patient with varying allele frequencies, shedding light on the genetic heterogeneity of PHL - Identified that genes involved in JAK/STAT, NFkB, and PI3K signaling, as well as antigen presentation, were frequently affected - Found *SOCS1* variants, mainly deletions, in most ctDNA, with many translocation breakpoints involving *SOCS1* - Revealed the origin of PHL HRS cells from partially selected germinal center B cells through VH-DH-JH rearrangement analysis - Correlated the amounts of pretherapy ctDNA with MTV, providing insight into tumor burden - Demonstrated that ctDNA was undetectable in patients with a favorable clinical course, while it remained detectable in those with an unfavorable clinical course
[47]	Capture NGS including 619 genes frequently mutated in cHL, other lymphomas and hematologic malignancies	Baseline ctDNA vs clinical outcome: AUC: 0.7832% (CI: 0.6383–0.9281) Minimum ctDNA VAF: 0.67% Median ctDNA VAF: 6.21% (95% CI, 3.86–10.57) Median ctDNA VAF responders: 8.72% Median ctDNA VAF non-responders: 2.9%	Plasma samples: - Diagnosis (*n* = 61) - On-treatment (*n* = 34) - Follow-up/Recurrence (*n* = 75)	- *STAT6* (34.43%) - *TNFAIP3* (31.15%) - *SOCS1* (24.49%) - *B2M* (22.95%) - Unique in Chinese individuals: *PCLO* (22.95%) and *LRP1B* (22.95%)	- Mutations in *B2M*, *TNFRSF14*, and *KDM2B* were associated with acquired resistance to treatment - ctDNA is identified as an informative biomarker for anti-PD-1 immunotherapy in refractory cHL patients
[48]	NGS Ampliseq panel including regions in the *NFKBIE*, *ITPKB*, *PTPN1*, *TNFAIP3*, *SOCS1*, *STAT6*, *B2M*, *XPO1* and *GNA13* genes	LOD VAF: ≥ 0.5%	Plasma samples: - Diagnosis (*n* = 60) - On-treatment (*n* = 55) - Follow-up/Recurrence (*n* = 2)	- *SOCS1* (50%) - *B2M* (33.3%) - *TNFAIP3* (31.7%) - *STAT6* (23.3%) - *ITPKB* (23.3%) - *NFKBIE* (13.3%) - *GNA13* (13.3%) - *XPO1* (10%) - *PTPN1* (5%)	- Developed a targeted NGS panel for the fast analysis of nine commonly mutated genes in biopsies and ctDNA of cHL patients using AmpliSeq technology - Included patients with a median age of 33.5 years, and identified variants in 70% of patients, with mutations in several genes including *NFKBIE*, *TNFAIP3*, *STAT6*, *PTPN1*, *B2M*, *XPO1*, *ITPKB*, *GNA13*, and *SOCS1* - Found that ctDNA concentration and genotype are correlated with clinical characteristics and presentation of cHL - Analysed ctDNA after C2, and it rapidly became undetectable in all cases - Concluded that variant detection in ctDNA is suitable for depicting the genetic features of cHL at diagnosis and may help assess early treatment response, especially in conjunction with PET
[49]	Three versions of customized RNA baits designed with SureSelect platform (Agilent)	Positive detection in spike-in samples with 0.5% of tumor purity	Plasma samples: - Diagnosis (*n* = 121) - Follow-up/Recurrence (*n* = 77)	- *TTN*, *SOCS1*, *TNFAIP3*, *ITPKB*, *STAT6*, *GNA13*, *B2M* and *CSF2RB* - Recurrent gains: 19p13.2 (65%); 2q31.2 (62%); 12q13.3 (*STAT2*, *STAT6*) (62%); 2p16.1 (*REL*) (61%); and 9p24.1 (*JAK2*, *CD274*) (55%) - Recurrent losses: 6q23.3 (*TNFAIP3*, *ECT2L*) (61%); 9q13 (59%); 13q32.3 (59%); 6q22.31 (58%); and 15q15.1 (57%)	- The study presents an integrated landscape of mutations and CNAs in HL - Several genotypes were linked to HL phenotypes and patient outcomes - Repeat cfDNA sequencing allowed for the assessment of MRD, which predicts treatment response and PFS - MRD assessment by cfDNA sequencing, even as early as a week after treatment initiation, provides valuable information for treatment guidance and relapse prediction
[50]	Ultra-deep NGS targeting 121 lymphoma-related genes	LOD VAF: 0.01%	Plasma samples: - Diagnosis (*n* = 6)	- *SMC3* (100%) - *TNFAIP3* (50%) - *TP53* (50%)	- Genetic alterations were identified in ctDNA samples, with a median of six variants per sample - Correlation with Clinical Indices: Association with mutations in necroptosis, metabolism and cell cycle occurrence. The genetic variation in ctDNA samples was significantly correlated with clinical indices in lymphoma patients, suggesting a potential link between genetic mutations and clinical parameters - Genetic heterogeneity was observed in different lymphoma subtypes, including HL
[51]	Capture-targeted NGS panel of 37 genes and five hot-spot areas of five genes panel design Targeted NGS panel of 42 genes	LOD VAF of 0.5%	Plasma samples: - Diagnosis (*n* = 60)	- *SOCS1* (28%) - *IGLL5* (26%) - *TNFAIP3* (23%) - *GNA13* (23%) - *STAT6* (21%) - *B2M* (19%), - *ARID1A*, *CSF2RB, KMT2D*, *ITPKB, PTPN1*, *EP300* Most recurrent variants: - *STAT6* (c-1249A > T, *n* = 4) and *XPO1* (c.1711G > A, *n* = 3)	- A total of 277 variants were detected in 73.5% of the samples with good-quality ctDNA. The median number of variants per patient was five, with a median VAF of 4.2% - Genotyping revealed somatic variants in genes including *SOCS1* (28%), *IGLL5* (26%), *TNFAIP3* (23%), *GNA13* (23%), *STAT6* (21%), and *B2M* (19%) - Several poor prognosis features, such as high LDH, low serum albumin, B-symptoms, IPI ≥ 3, or advanced stage, were associated with higher amounts of ctDNA
[52]	CAPP-seq panel covering regions of 77 recurrently mutated genes in mature B-cell tumors	Sensitivity of biopsy-confirmed tumor mutations detected in ctDNA: 87.5% (CI: 95%, 79.2–92.8%) Mean VAFs in ctDNA: 5.5% (range 0.29%-74%)	Plasma samples: - Diagnosis (*n* = 80) - On-treatment (*n* = 24) - Follow-up/Recurrence (*n* = 32) - After failing autotransplant salvage (*n* = 6) - After failing brentuximab vedotin (*n* = 5) - Before and during therapy with nivolumab (*n* = 5)	*- STAT6* (37.5%) *- TNFAIP3* (35%) *- ITPKB* (27.5%) *- NF-KB* (46.2%), *PI3K/AT* (46.2%) - Cytokine signaling (37.5%) - Epigenetic genes were cumulatively affected (35%) - Genes involved in immune surveillance (37.5%) *- NOTCH* (20%)	- Use of ctDNA for Genomic Analysis: Successful identification of cHL genetics using a highly sensitive next-generation sequencing approach for ctDNA - ctDNA mirrors Hodgkin and Reed-Sternberg cell genetics - *STAT6* identified as the most frequently mutated gene in approximately 40% of cases - Longitudinal ctDNA profiling reveals treatment-dependent patterns of clonal evolution in relapsing patients and those under immunotherapy - ctDNA changes during therapy can track residual disease, potentially aiding in early identification of chemorefractory patients with cHL - Patients maintaining partial response under nivolumab, ancestral clones were suppressed and replaced by novel harboring new mutations - Patients achieving complete response had a larger drop in ctDNA load after two ABVD courses compared with relapsing patients - Relapsing patients who were inconsistently judged as interim PET/CT negative had a less than 2-log drop in ctDNA
[54]	Ultra low-pass sequencing for CNAs detection in plasma	Patients vs healthy controls: Sensitivity: 89.3% Specificity: 87.2%	Blood samples Plasma samples: - Diagnosis (*n* = 177) - On-treatment (*n* = 132–136)	- Gain of 15q21.3-q26.3 - Loss of 3p26.3-p13 and 12q21.31-q24.33	- Over 90% of cHL patients exhibited CNAs in cfDNA - Gains encompassed 2p16 (69%), 5p14 (50%), 12q13 (50%), 9p24 (50%), 5q (44%), 17q (43%), and 2q (41%). Losses mostly affected 13q (57%), 6q25-q27 (55%), 4q35 (50%), 11q23 (44%), and 8p21 (43%) - Novel recurrent CNAs identified in cHL included loss of 3p13-p26 and 12q21-q24 and gain of 15q21-q26 - ctDNA concentration at diagnosis was associated with advanced disease, male sex, extensive nodal disease, elevated ESR, MTV, and HRS cell burden - CNAs and ctDNA concentrations rapidly diminished upon treatment initiation - Persistence of CNAs was associated with an increased probability of relapse
[55]	Shallow whole-genome sequencing	HL cases had a significantly elevated number of plasma EBV DNA Sensitivity of EBV detection: 100% Specificity of EBV detection: 82.6% Sensitivity using CNAs: 84.2% Minimum CPA: 0.64	Plasma samples: - Staging (*n* = 44) - On-treatment (*n* = 22) - Follow-up (*n* = 4)	- 2p16.1 gains (*REL*), 9p24.1 gains (*JAK2* and *PD-L1*)	- The detection of CNAs in blood has the potential to differentiate between DLBCL and HL - At the time of diagnosis, liquid biopsies detected CNAs in 84.2% of HL patients (88.6% in classical HL) - HL patients had higher-amplitude CNAs in their liquid biopsies compared to tissue biopsies, suggesting that tumor DNA is more abundant in plasma - Elevated plasmatic EBV DNA fragments were found in 39.5% of HL, achieving a sensitivity of 100% compared to the current standard - Longitudinal analysis revealed that when detectable, copy number patterns were similar across different staging moments in refractory/relapsed patients - The overall profile anomaly is highly correlated with the total MTV
[56]	PhasED-seq	LOD VAF of > 0.0001%	Plasma samples: - Diagnosis (*n* = 29) - On-treatment (*n* = 26) - End-of-treatment (*n* = 24)		- Decrease in ctDNA levels is associated with superior PFS when measured after cycle two and at the end of treatment
[57]	RT-qPCR for *POLR2* gene	Diagnosis cfDNA mean patients aged ≤ 10 years: 14 ng/mL Diagnosis cfDNA mean patients aged > 10 years: 114 ng/mL	Plasma samples: - Diagnosis (*n* = 43)		- High cfDNA at HL Diagnosis: Elevated cfDNA levels in HL patients at diagnosis - < 10-year higher cfDNA level - Plasmatic cfDNA could be used as a prognostic predictor for PHL patients
[58]	RT-qPCR for *POLR2* gene	Diagnosis cfDNA mean value: 112 ng/mL Unselected subgroup of cHL cohort: Diagnosis cfDNA mean: 40 ng/mL TP1 cfDNA mean: 22 ng/mL TP2 cfDNA mean: 22 ng/mL TP3 cfDNA mean: 15 ng/mL	Plasma samples: - Diagnosis (*n* = 155) - On-treatment: - TP1 (*n* = 75) - TP2 (*n* = 41) - TP3 (*n* = 25)		- Elevated cfDNA levels in cHL patients at diagnosis - Median cfDNA levels decrease during chemotherapy treatment - cfDNA at diagnosis is correlated with a diffuse inflammatory status - An increase in cfDNA after the first chemotherapy cycle is associated with a worse prognosis and mediastinal bulky involvement in cHL patients
[59]	RT-qPCR targeting BAMH1W (EBV) and β-globin (human) regions	LOD EBV: 5 copies/mL Median cfDNA in patients with HL at diagnosis: 434 ng/mL (range 2.3–17,306) Median cfDNA in patients with HL patients without recurrence (at diagnosis): 406 ng/mL Median cfDNA levels in patients with HL presenting recurrence (at diagnosis): 569 ng/mL	Plasma samples: - Diagnosis (*n* = 34) - On-treatment (*n* = 34) - Follow-up/Recurrence (*n* = 5)		- Investigated the potential roles of serum EBV DNA and cfDNA as markers for prognosis and treatment response in PHL and NHL) - Serum EBV DNA copy numbers were elevated at initial diagnosis in a significant number of HL and NHL cases - Both serum EBV DNA copy numbers and cfDNA levels decreased significantly after induction treatment and during follow-up - No significant differences were detected in median cfDNA levels based on disease stages, response status to treatment, or presence of recurrent disease - Serum EBV DNA copy numbers and cfDNA levels may serve as informative markers, with initial elevations that decrease with treatment response
[60]	NGS Ampliseq panel including regions in the *NFKBIE*, *ITPKB*, *PTPN1*, *TNFAIP3*, *SOCS1*, *STAT6*, *B2M*, *XPO1* and *GNA13* genes	LOD VAF: ≥ 0.5% (the same NGS panel as reference 48)	Plasma samples: - Diagnosis (*n* = 48)		- Burden parameters TMTV, TMTS and TLG were significantly associated with ctDNA concentration in cHL - Association between dispersion parameters TumBB and Dmax and ctDNA concentration
[61]	CAPP-seq targeting region of 133 genes recurrently mutated in B-cell lymphomas	Baseline ctDNA and iPET for outcome prediction: Sensitivity: 70.6% Specificity: 94.7% LOD VAF: 0.1%	Plasma samples: - Diagnosis (*n* = 55) - On-treatment (*n* = 45 and 34)	- *STAT6* (44%) - *B2M* (38%) - *TNFAIP3* (36%) - *GNA13* - *SOCS1* (31%) - *ITPKB* (29%) - *XPO1* (22%) - *TP53* - *PTPRD* (18%) - *BTG1* (16%)	- Confirmation that recurrence cHL patients are predominantly mutated in NF-kB, JAK-STAT and PI3K-Akt pathways - Recurrence cHL patients with higher baseline ctDNA levels are associated with treatment failure within 18 months of chemotherapy initiation - ctDNA is a useful tool that complements iPET to monitor MRD during treatment and for clinical decisions for r/r cHL patients

This table compiles studies involving the utilization of circulating DNA. Percentages in the “Altered genes/genomic regions” column indicate the proportion of samples/patients carrying mutations in a specific gene

*ABVD* Adriamycin, bleomycin, vinblastine, dacarbazine*, AUC* Area under the curve*, C2* after two cycles of chemotherapy, *CAPP-seq* Cancer Personalized Profiling-sequencing, *cfDNA* cell-free DNA *cHL* Classical Hodgkin Lymphoma, *CI* confidence interval, *CAN* Copy Number Alteration, *CPA* Copy Number Profile Abnormality score, *ctDNA* circulant tumor DNA, *ddPCR* digital-droplet PCR, *DLBCL* Diffuse large B cell lymphoma, *Dmax* distance between the 2 lesions that are farthest apart, *EBV* Epstein-Barr Virus, *ESR* Erythrocyte Sedimentation Rate, *HC-tNGS* Hybrid capture targeted-NGS, *HL* Hodgkin Lymphoma, *HRS* Hodgkin-Reed Sternberg, *Indels* insertions and deletions, *iPET* interim Positron emission tomography, *IPI* International prognostic index, *LDH* Lactate dehydrogenase, *LOD* Limit of detection, *MRD* Minimal Residual Disease, *MTV* Metabolic Tumor Volume, *NGS* Next-Generation Sequencing, *NHL* non-Hodgkin lymphoma, *PET* Positron emission tomography, *PET/CT* Positron emission tomography/Computed Tomography, *PFS* progression-free survival, *PhasED-seq* Phased variant enrichment and detection sequencing, *PHL* paediatric Hodgkin lymphoma, *r/r* relapsed or refractory, *RT-qPCR* reverse transcription quantitative polymerase chain reaction, *SABER* sequence affinity capture and analysis by enumeration of cell-free receptors, *SNVs* single nucleotide variants, *TLG* total lesion glycolisis, *TMTS* total metabolic tumorsurface, *TMTV* total metabolic tumor volume, *TP1* after first chemotherapy cycle, *TP2* after stop chemotherapy, *TP3* after radiotherapy, *TumBB* volume of the bounding box including the tumors, *VAF* Variant Allele Frequency

### Beyond circulating tumor DNA: extracellular vesicles and circulating RNAs

Extracellular vesicles (EVs) exhibit a wide range of sizes and biogenesis pathways. Apoptotic bodies (1–4 µm) and microvesicles (100–1000 nm) originate from plasma membrane budding. In this regard, exosomes, a subset of EVs, are small membrane microvesicles (40–150 nm) derived from endosomes within multivesicular bodies [64] playing a crucial role in intercellular communication. The formation of exosomes begins with the invagination of the plasma membrane, leading to the creation of intracellular multivesicular bodies containing intraluminal vesicles. Exosomes report tumor-derived information as they carry diverse types of biomolecules, including proteins, lipids, and nucleic acids which are essential for molecular interrogation. Their vital role in intercellular communication has been extensively documented [65, 66]. Importantly, exosomes have garnered significant attention due to their remarkable capacity to transport molecules of interest, which hold potential as disease biomarkers and tumor-derived information [67].

Furthermore, EVs have been found to influence neoplasia promotion. Studies have revealed that various molecules, such as nucleic acids and signaling proteins, can induce protumorigenic effects in tumor microenviroment [64]. Metastasis research has also highlighted the involvement of EV-mediated communication, for example in prostate cancer [68, 69]. In the field of biomedical research, EVs are being extensively investigated as potential biomarkers for predicting clinical outcomes. Their intriguing properties and ability to reflect disease-related changes make them promising candidates for future diagnostic and prognostic applications.

In HL, one of the earliest studies showed that these patients present a more prominent appearance of smaller EVs (< 0.3 µm) than the control group. Also, the concentration of plasmatic EVs was statistically higher in patients with HL than in the control group. Importantly, some tumor-related antigens significantly expressed in HL compared to controls were CD61 and CD30. In particular, CD30 was observed with higher levels in early disease stages [70].

Proteins are among the components present in EVs. In this regard, Repetto et al. carried out a proteomic study using two-dimensional difference gel electrophoresis followed by liquid chromatography-tandem mass spectrometry to identify proteins in EVs from patients with HL. They described differences in certain proteins between relapsed and no-relapsed pediatric patients with HL. Importantly, the proteins found exclusively in patients when comparing with healthy controls were described to participate in platelet degranulation and serine-type endopeptidase activities [66]. This study is summarized in Table 2 and included in Fig. 2.

**Table 2:** Protein profiles of exosomes

Reference	Method employed	Observed detection value	Samples	Proteins	Main findings
[66]	2D-DIGE followed by liquid chromatography–tandem mass spectrometry	Differentially abundant: |fold change|≥ 1.5, *p* < 0.05	Plasma samples: - Diagnosis (*n* = 15)	Non relapsed: C4-A, C4B, FGG, ITIH2 and IGHM Relapsed: APOA1, APOA4, CLU, HP, ORM1 and TTR	- Identification of a subset of 11 plasma-derived EV proteins whose levels at the time of the pediatric HL diagnosis were related to the presence or absence of relapse - Five proteins were more abundant in non-relapsed HL and six were more abundant in relapsed cases - Determination of 89 newly discovered proteins to be related to paediatric HL which were enriched in complement activation, classical pathway and antigen binding - High levels of FGG in non-relapsed pediatric HL at diagnosis

This table includes a summary of a study on EVs proteins

*2D-DIGE* Two-dimensional difference gel electrophoresis, *APOA1* apolipoprotein A-I, *APOA4* apolipoprotein A-IV, *C4-A* isoform 2 preproprotein of complement C4-A, *C4B* complement C4-B, *CLU* clustering, *EV* extracellular vesicle, *FGG* fibrinogen γ chain, *HL* Hodgkin lymphoma, *HP* haptoglobin, *IGHM* immunoglobulin heavy chain constant region mu, *ITIH2* inter-α-trypsin inhibitor heavy chain H2, *LOD* Limit of detection, *ORM1* α-1-acid glycoprotein 1, *TTR* transthyretin

Other intriguing investigations have focused on developing novel biosensors to detect EVs released by HRS cells, with potential implications for the diagnosis and treatment-response monitoring of this disease. In this context, Slyusarenko et al. employed the HRS marker CD30 to capture EVs from HRS cells using gold nanoparticles (AuNPs) with peroxidase activity. They observed an increased number of CD30-positive particles in the plasma of patients with cHL compared to healthy individuals. Furthermore, they discovered a strong correlation with PET-CT scans and a significant decrease in the number of CD30-positive particles in patients with cHL after two cycles of chemotherapy [71].

Furthermore, EVs carry a wide array of cargo with significant biological relevance in cancer. Among these cargoes, microRNAs (miRNAs) are small, double-stranded RNA molecules comprising approximately 19–35 nucleotides. They play a crucial role in regulating gene expression, controlling differentiation, and modulating proliferation at the post-transcriptional level. Notably, miRNAs exhibit remarkable stability in the bloodstream [72]. Recent studies on exosome-derived miRNAs in various tumor types have indicated their potential use as biomarkers. These biomarkers, in combination with other circulating RNAs, can serve diagnostic purposes and also aid in predicting responses to treatments [73, 74].

In a groundbreaking analysis involving patients with cHL, Van Eijndhoven et al. investigated the association between EV-associated and free plasma miRNAs with metabolic disease. The authors observed a more extensive repertoire of EV-associated miRNAs and identified elevated levels of miR24-3p, miR127-3p, miR21-5p, let7a-5p, and miR155-5p in patients with cHL compared to healthy controls. Importantly, this study demonstrated the potential of miRNAs for disease monitoring. They observed that miRNA levels decreased in patients achieving complete metabolic response during long-term plasma follow-up, consistent with FDG-PET findings. Additionally, these levels increased in relapsed patients [75].

As previously mentioned, conventional imaging techniques for evaluating treatment response in Hodgkin lymphoma (HL) cannot be frequently repeated, highlighting the need for identifying novel biomarkers for monitoring therapy outcomes. In a recent study, Drees et al. conducted a comparison between the expression of specific EV-associated miRNAs and FDG-PET assessments in patients with cHL. Their findings revealed a significant increase in miR-127-3p, miR-155-5p, miR-21-5p, miR-24-3p, and let-7a-5p in pre-treatment patients with cHL compared to individuals who exhibited treatment response. Remarkably, these miRNA levels remained elevated in non-responsive patients. Furthermore, combining EV-miR-127-3p and/or EV-let-7a-5p with serum TARC (a validated protein biomarker in cHL) significantly enhanced the accuracy of predicting PET status, resulting in a specificity of 83.8% to 85.0% and a sensitivity of 93.5%, with a negative predictive value of 96% [76].

In this context, the same investigators also examined the relationship between blood-based biomarkers, including EV-miRNAs, and specific assessments using FDG-PET. Prior to treatment initiation, they observed correlations between EV-miR127-3p, EV-miR24-3p, serum TARC, and complete blood counts with the metabolic tumor volume and dissemination features, albeit not with intensities. Additionally, certain other EV-miRNAs exhibited weak correlations with other PET features [77]. In biofluids, miRNAs can circulate either in combination with EVs, as previously mentioned, or as free molecules, often associated with proteins. To our knowledge, there is only one significant publication that characterizes the presence of free miRNAs in cHL. In this study, conducted by Jones et al., over 1,000 miRNAs were profiled in a small cohort comprising 14 primary cHL tissues and eight healthy lymph nodes. The findings revealed an association between miR-494 and miR-1973 with the disease. Subsequently, the presence of these miRNAs was assessed in the plasma of a cohort of patients. Blood samples were analyzed at various time points, including pre-treatment, during treatment, and after remission. The results showed that miR-494, miR-1973, and miR-21 exhibited increased levels in patients with cHL compared to healthy controls, and these levels became undetectable after achieving remission. Notably, only miR-494 and miR-1973 correlated with interim therapy responses [78].

Another critical aspect to consider is the potential for therapy-related side effects, often associated with the toxicity mentioned earlier. In this context, a recent study delved into the impact of therapy on fertility, specifically examining the risk of temporary or permanent loss of fertility. In this study, conducted by Caponnetto et al., researchers utilized follicular fluid samples from women affected by HL. Their findings revealed the deregulation of 13 miRNAs in these women when compared to the control group. Several of these deregulated miRNAs were found to play roles in biological processes linked to follicle development and oocyte maturation [79].

All of the aforementioned studies have underscored the promising potential of liquid biopsy, extending beyond ctDNA, in the context of cHL. However, further investigations are warranted, particularly those incorporating high-throughput sequencing technologies, to provide a comprehensive overview of the circulating RNA landscape. Furthermore, future studies that integrate insights from various 'omics' disciplines have the potential to yield innovative non-invasive tools for monitoring treatment responses.

These studies are summarized in Table 3 and included in Fig. 2.

**Table 3:** Beyond circulating tumor DNA: Extracellular vesicles and circulating miRNAs

Reference	Method/s employed	Observed detection values, LOD, Sensitivity, Specificity, etc	Samples	miRNA expression	Main findings
[75]	Small RNAseq	AUC of miR127-3p for diagnosis: 0.80 Sensitivity: 1–10% Minimum read counts for miRNA detection: ≈1 × 10^2^ summed counts	Plasma samples: - Diagnosis (*n* = 20) - On-treatment (*n* = 7)	**miR127-3p ↑, miR155-5p ↑, miR21-5p ↑, let7a-5p ↑, miR24-3p ↑**	- Decrease in the abundance of five miRNAs cHL related panel associated with CMR to treatment - miR21-5p, miR127-3p, let7a-5p, miR24-3p, and miR155-5p associated to EV were elevated in primary and relapsed cHL patients compared to healthy individuals - miRNA analysis in EV fractions by RNAseq can increase the signal-to-noise ratio of miRNA biomarkers in plasma
[76]	Small RNAseq PE150 Multiplex stemloop qRT‐PCR	miRNAs panel to stratify PET-positive from PET-negative patients AUC: 0.855 (CI 0.781–0.929) miR-127-3p and miR24-3p to stratify PET-positive from PET-negative patients AUC of 0.872, 79% specificity and 80% sensitivity Minimum counts per million: 1LogCPM	Plasma samples: - Diagnosis (*n* = 26) - On-treatment (*n* = 73) -Follow-up/Recurrence (*n* = 94)	**miR-127-3p ↑**, **let-7a-5p ↑**, **miR-21-5p ↑,** miR-10b-5p ↓, miR-150-5p ↓, **miR-155-5p ↑, miR-24-3p ↑**	- A differential miRNA signature of 33 miRNAs in active (PET-positive) disease compared to CMR - miR-155-5p and miR-24-3p were overexpressed but it reached significance only by RT-qPCR
[77]	Multiplex stem loop RT-qPCR	-	Plasma samples: - Diagnosis (*n* = 30)	**miR127-3p ↑, miR155-5p ↑**	- Two miRNAs were correlated with higher number of lesions
[78]	qRT-PCR	Cutoff values: - miR-494: 3.0 × 10^5^ copies/µL plasma , 85% sensitivity and 60% specificity - miR-1973: 1.6 × 10^6^ copies/μL plasma, 75% sensitivity and 67% specificity - miR-21: 1.0 × 106 miR-21 copies/μL plasma, 95% sensitivity and 86% specificity	Plasma samples: - Diagnosis (*n* = 42) - On-treatment (n = 38 and *n* = 37)	**miR-494 ↑, miR-1973 ↑, miR-2861 ↑, miR-638 ↑, miR-663b ↑, miR-16 ↑**	- A signature of 238 miRNAs overexpressed was shown - They observed 50 differentially expressed human miRNAs - Selection of top five miRNAs with increased expression
[79]	High-throughput micro-RNA expression (NanoString nCounter system)	Minimum Log2 Normalized counts detected: ≈ 3	Follicular fluid samples: - Diagnosis (*n* = 23)	**miR-1285-5p ↑, miR-1303 ↑, miR-1972 ↑, miR-2117 ↑, miR-4455 ↑, miR-548ah-5p ↑, miR-574-5p ↑, let-7b-5p ↓, miR-3195 ↓, miR-371a-5p ↓, miR-423-5p ↓, miR-4532 ↓, miR-503-5p ↓**	- Two sets of miRNAs signatures, 27 and 14 differentially expressed miRNAs using different statistical analyses

This table includes the published research involving the utilization of circulating miRNAs. Arrows up and down represent increased or decreased levels of that miRNAs respectively. miRNAs highlighted in bold have also been validated through RT-qPCR

*AUC* Area under the curve, *cHL* Classical Hodgkin Lymphoma, *CI* confidence interval, *CMR* complete metabolic response, *CPM* Counts per million, *EV* Extracellular vesicles, *LOD* Limit of detection, *PET* Positron emission tomography, *RT-qPCR* reverse transcription quantitative polymerase chain reaction

### Beyond circulating tumor DNA: Cytokines

Cytokines, characterized by a relative molecular weight below 30,000 Da, are either polypeptides or glycoproteins. They play a crucial role in supplying signals for the growth, differentiation, inflammation, or anti-inflammatory responses of various cell types. Additionally, they have the capacity to activate immune cells against tumors or counteract immunosuppression, ultimately leading to the inhibition of tumor growth [80]. As mentioned above, HRS cells in cHL constitute a minor fraction of the tumor and are overshadowed by a predominant mixed inflammatory infiltrate. Patients with cHL often exhibit constitutional symptoms such as fever, weight loss, and night sweats, along with a discernible systemic deficiency in cell-mediated immune responses. These distinct clinical and histopathologic characteristics of cHL are indicative of an abnormal immune response, primarily attributed to a diverse array of cytokines produced by HRS cells and, to a lesser extent, by the surrounding reactive infiltrate [81].

Similar to ctDNA, cytokines can be identified in blood samples from cancer patients through minimally invasive approaches [82]. Their presence has been demonstrated to correlate with the risk of developing specific types of cancer [83–85] as well as being associated with the tumor stage [86] and prognosis [87] or influencing treatment efficacy [88]. Indeed, certain studies have conducted a combined analysis to detect both ctDNA and cytokines as potential non-invasive biomarkers [89, 90]. In cHL, one interesting study assessed the prognostic value of pretreatment serum cytokine levels in cHL. The authors observed elevated levels of twelve cytokines in patients with cHL compared to controls, with HGF, IL-6, IL-2R, IP-10, and MIG linked to poorer event-free survival (EFS). IL-2R and IL-6 were independently prognostic, associating with a higher risk of early relapse and death. Notably, this remained significant after adjusting for the International Prognostic Score (IPS). The study suggests that pretreatment cytokine profiling, particularly focusing on IL-6 and IL-2R, could effectively identify high-risk cHL patients prone to early-disease relapse and may serve as an additional prognostic tool beyond existing risk stratification methods [91]. In addition, other publications have also confirmed the role of IL-6 as biomarker for disease outcome in patients with cHL [92]. Furthermore, another study showed the involvement of other cytokines such as IL-10, TNF-α, IFN-γ, IL-8, and TNFSF10 associated with high-risk disease, as well as CCL13, IFN-λ1, and IL-8 with treatment response [93]. Additionally, a recent publication showed serum concentration kinetics of key cytokines in patients with cHL. They compared these levels with PET/CT scan results and treatment outcomes. Herein, they investigators observed the median concentration of IL-10, IL-6, TNF-α at end-of-treatment-PET (EOT-PET) decreased in comparison with the levels at initial PET (PET-0), while IFNγ and IPI10 showed an increase. Also, IL-8 levels were increased in HL compared to FL. Overall, they observed that only the levels of TARC showed variations during therapy, correlating well with the results of PET/CT and therefore also with the response to therapy, especially in cHL. These results should be taken with caution considering the small patient cohort included in this study [94]. However, TARC levels prove useful in assessing treatment failure or extrapulmonary spread, with decreased levels correlating with better treatment response and complete remission, while elevated levels are associated with positive PET scans in other studies [95, 96]. Finally, other investigations have linked viral infections as a crucial factor influencing local expression of chemokines rather than HL subtypes [97–99].

In cHL, cytokines may play a dual role—complementing ctDNA or other tumor components in disease diagnosis, treatment monitoring, or MRD detection, as observed in studies on other tumor types [89, 90] and contributing to the development of novel treatment strategies [80, 100–102]. Cytokines, with their pivotal signaling role in cHL, stand out as promising components in the quest for biomarkers through liquid biopsies.

The previously mentioned studies are summarized in Table 4 and included in Fig. 2.

**Table 4:** Beyond circulating tumor DNA: Cytokines

Reference	Method/s employed	Observed detection values: LOD, Sensitivity, Specificity, etc	Samples	Cytokines/Protein	Main findings
[85]	ELISA	LOD sCD30: 6 units/mL LOD IL-6: 0.2 pg/mL LOD IL-10: 2 pg/mL	Serum samples: - Diagnosis (*n* = 103)	sCD30↑ and IL6↑ IL-10↑ (EBV positive cases)	- sCD30 and IL6, and detectable IL10 were elevated previous to cHL diagnosis
[91]	ELISA	Median EGF values: 142.2 (range 10.0–1,002.8) Median FGF values: 11 (range 11.0–201.7) Median G-CSF values: 171.9 (range 10–1,383.2) Median HGF values: 717.0 (range 50.0–3075.8) Median Il-6 values: 13.1 (range 1.5–1429.6) Median IL-8 values: 36.5 (range 1.5–13,333.0) Median IL-12p40 values: 262.4 (range 96.2–2386.4) Median IL-2R values: 1,188.5 (range 183.2–26,670.7) Median IL-10 values: 67.8 (range 13.7–2322.7) Median MIG values: 98.9 (range 3.0–2555.0) Median TNF-α values: 2.5 (range 2.5–332.9) Median VEGF values: 12.0 (range 2.5–56.3)	Serum samples: - Diagnosis (*n* = 140)	EGF ↑, FGFb↑, GCSF ↑, HGF↑, IL-6↑, IL-8↑, IL-12↑, IL-2R↑, IP-10↑, MIG↑, TNF-α↑, VEGF↑	- Higher HGF, IL-6, IL-2R, IP-10, MIG levels were associated with worst EFS - IL-2R and IL-6 were independently prognostic and were related with higher risk of relapse and death. These elevated cytokines correlated with IPS, sCD30 and TARC levels
[92]	ELISA	IL-6 prediction for overall survival: - Sensitivity: 79% - Specificity: 69% - Median serum IL-6: 5.0 pg/mL (range 0.4–314)	Serum samples: - Diagnosis (*n* = 88)	IL-6** ↑**	- IL-6 correlated with increasing ESR, decreasing serum albumin and increasing age - IL-6^+^ leukocytes are an independent biomarker for inferior EFS and OS - Serum IL-6 is not associated with IL-6^+^ leukocytes and IL-6^+^ HRS cells
[93]	Luminex® platform	Mean TGF-α concentration: 6.4 ± 3.5 pg/mL Mean CXCL13 concentration: 7.5 ± 1.8 pg/mL Mean IL-10 concentration: 3.8 ± 2.4 pg/mL Mean CXCL9 concentration: 12.0 ± 2.3 pg/mL Mean CCL19 concentration: 8.8 ± 1.4 pg/mL Mean CCL17 concentration: 10.0 ± 1.4 pg/mL Mean IL-6.2 concentration: 3.4 ± 2.1 pg/mL Mean IL-6.3 concentration: 3.4 ± 2.4 pg/mL Mean GDF-2 concentration: 4.8 ± 1.9 pg/mL Mean HB-EGF concentration: 3.7 ± 1.5 pg/mL Mean Endothelin-1 concentration: 4.2 ± 1.4 pg/mL	Plasma samples: - Diagnosis (*n* = 56)	TGF-α↑, CXCL13↑, IL-10↑, CXCL9↑, CCL19↑, CCL17↑, IL-6↑, IL-6.2↑, IL-6.3↑, GDF-2↓, HB-EGF↓, Endothelin-1↓	- 32 cytokines/chemokines were differentially expressed compared to controls - High risk patients presented higher levels of IL-10, IL-8.2, IFN-γ, TNF-α - CCL13, IFN-γ and IL-8 are elevated in slowly responders compared to rapid responders to therapy - TNFSF10 was elevated in relapsed patients and was associated with worse EFS
[94]	Simple Plex system	Mean serum cytokines levels at PET-0: - IL-10: 4.75 pg/mL - IL-8: 6.98 pg/mL - IL-6: 7.69 pg/mL - TNF-α: 12.66 pg/mL - IFN- γ: 4.15 pg/mL - IPI10: 125.9 pg/mL - TARC: 8327 pg/mL Mean serum cytokine levels at iPET: - IL-10: 1.98 pg/mL - IL-8: 10.74 pg/mL - IL-6: 2.543 pg/mL - TNF-α: 11.4 pg/mL - IFN- γ: 7.93 pg/mL - IPI10: 211.8 pg/mL - TARC: 591.16 pg/mL Mean serum cytokine levels at EOT-PET: - IL-10: 3.675 pg/mL - IL-8: 12.58 pg/mL - IL-6: 4.822 pg/mL - TNF-α: 9.8 pg/mL - IFN- γ: 10 pg/mL - IPI10: 206 pg/mL - TARC: 525 pg/mL	Serum samples: - Diagnosis (*n* = 6) - On-treatment (*n* = 6) - End of treatment (*n* = 6)	EOT-PET vs PET-0: IL-10↓, IL-6↓, TNF-α↓, IL-8↑, IFN- γ↑, IPI10↑, TARC↓ iPET vs PET-0: TARC↓	- IL-8 increased in DLBCL and HL, while decreased in FL - TARC values significantly lower at iPET in HL patients - Correlation between TARC levels and PET/CT results observed in all subtypes - Almost all patients achieved CR at EOT-PET based on Deauville score - TARC values demonstrated consistent correlation with PET/CT results and treatment response, especially in HL patients
[95]	ELISA and Simple PLEX system	Before brentuximab vedotin - Median IL-6: 2.27 pg/mL (range 0.10–154) - Median IL-10: 0.38 pg/mL (range 0.09–112) - Median TNF-α: 2.55 pg/mL (range 0.55–15.15) - Median IFN-γ: 8.66 pg/mL (range 1.45- 554) - Median TARC: 6852 pg/mL (range 164–163,169) After brentuximab vedotin - Median IL-6: 1.41 pg/mL (range 0.09–34) - Median Il-10: 0.45 pg/mL (range 0.14–18) - Median TNF-α: 2.25 pg/mL (range 0.58–22) - Median IFN-γ: 9.01 pg/mL (range 2.62–113) - Median TARC: 875 pg/mL (range 151–34,770)	Serum samples: - Diagnosis IL-6, IL10, TNF-α and IFN-γ (*n* = 37) - Diagnosis TARC (*n* = 64) - On-treatment IL-6, IL10, TNF-α and IFN-γ (*n* = 37) - On-treatment TARC (*n* = 57)	TARC↑, IL-6↑, IL-10↑, TNF-α↑ and IFN-γ↑	- TARC could be used as biomarker to predict EFS - Patients with extra nodal disease have elevated IFN-γ and IL-10 - Patients with B symptoms had increased levels of TNF-α and IL-10 - TARC was the only cytokine that significantly decrease after treatment
[96]	ELISA	Median TARC values at baseline: 37,790 pg/mL (range 13,920–71920) Median TARC-2 values: 438 pg/mL (range 275–731) TARC-2 levels for outcome prediction: - Sensitivity: 43% - Specificity: 82%	Serum samples: - Diagnosis (*n* = 266) - On-treatment (*n* = 266) - Follow-up (*n* = 266)	TARC **↑**	- TARC levels were significantly higher positive PET-2 patients -2 and treatment failure than those PET-2 negative and in complete remission - Worse prognosis in patients with negative PET-2 and TARC-2 > 800 pg/mL
[98]	ELISA	Median IL-10 at diagnosis: 22.4 pg/mL (IQR 11–2-54.4) Median IL-6 at diagnosis: 23.5 pg/mL (IQR 5.2–128.7) Median BAFF at diagnosis: 898.9 pg/mL (IQR 720.2–1790.3)	Serum samples: - Diagnosis (*n* = 83) - On-treatment (*n* = 38) - Follow-up (*n* = 38)	IL-10↑, Il-6↑, BAFF↑	- IL-10, IL-6, and BAFF levels were significantly higher in HIV-cHL patients compared to controls - Levels increased in advanced-stage lymphoma compared to limited-stage - Cytokine levels decreased after HIV-cHL diagnosis and treatment - Whole B-cell counts were similar in HIV-cHL patients and controls - Different distribution of B-cell subsets in HIV-cHL patients, with higher ratios of naive B-cells over memory B-cells - More marked accumulation of naive B-cells in patients with advanced cHL stages. During follow-up, total B-cell counts increased, and the proportion of naive B-cells increased further
[99]	ELISA	Mean MIP-1α levels in HL patients: 78.3 ± 299.5 pg/mL Mean MIP-1β levels in HL patients: 131.6 ± 209.8 pg/mL Mean IL-13 levels in HL patients: 6.1 ± 26.8 pg/mL	Serum samples: - Diagnosis (*n* = 53)	MIP-1α↑, MIP-1β↑, IL-13↑	- Increased expression of MIP-1α, MIP-1β and IL-13 correlated with EBV infection and LMP1 expression and were more common in patients aged > 60 years, and was associated with poorer prognosis - MIP-1α, MIP-1β and IL-13 levels are higher in HL patients compared to controls. These cytokines are increased in patients undergoing EBV infection
[102]	ELISA	–	Serum samples: - Diagnosis (*n* = 74)	HGF↑	- Elevated HGF levels is a potential biomarker for HL diagnosis and relapse - Suppressed ALC/AMC ratios and elevated IL-2R are independent predictors of overall survival - IL-2R is associated with peripheral blood surrogate markers of immunosuppression

This table encompasses the published research involving the utilization of circulating cytokines and proteins. Arrows up and down represent increased or decreased levels of a cytokine/protein respectively

*ALC/AMC* absolute lymphocyte count/absolute monocyte count, *BAFF* B-cell activating factor, *CCL13* Chemokine (C–C motif) ligand 13, *CCL17* Chemokine (C–C motif) ligand 17, *CCL19* Chemokine (C–C motif) ligand 19, *cHL* Classical Hodgkin Lymphoma, *CR* Complete Response, *CXCL9* Chemokine (C-X-C motif) ligand 9, *CXCL13* Chemokine (C-X-C motif) ligand 13, *DLBCL* Diffuse large B-cell lymphoma, *EBV* Epstein-Barr Virus, *EFS* Event-free survival, *EGF* epidermal growth factor, *ELISA* Enzyme-Linked Immuno Sorbent Assay, *EOT-PET* End-of-treatment PET, *ESR* Erythrocyte Sedimentation Rate, *FGFb* Basic fibroblast growth factor, *FL* follicular lymphoma, *GCSF* granulocyte-colony stimulating factor, *GDF-2* Growth/differentiation factor 2, *HB-EGF* Heparin-binding epidermal growth factor-like growth factor, *HGF* Hepatocyte Growth Factor, *HIV* human immunodeficiency virus, *HL* Hodgkin Lymphoma, *HRS* Hodgkin-Reed Sternberg, *IFN-γ* Interferon gamma, *IL-2R* Interleukin 2R, *IL-6* Interleukin 6, *IL-6.2* Interleukin 6.2, *IL-6.3* Interleukin 6.3, *IL-8* Interleukin 8, *IL-8.2* Interleukin 8.2, *IL-10* Interleukin 10, *IL-12* Interleukin 12, *IL-13* Interleukin 13, *iPET* interim Positron emission tomography, *IPI10* CXCL10/Chemokine (C-X-C motif) ligand 10, *IPS* International prognostic score, *IQR* Interquartile range, *LMP1* Latent membrane protein 1, *LOD* Limit of detection, *MIG* Monokine induced by gamma, *MIP-1α* macrophage inflammatory protein-1α, *MIP-1β* macrophage inflammatory protein-1β, *OS* overall survival, *PET-0* Diagnosis PET, *PET-2* PET after the first two treatment cycles, *PET/CT* Positron emission tomography/Computed Tomography, *sCD30* serum CD30, *TARC* Thymus and activation-regulated chemokine, *TARC-2* TARC after two ABVD cycles, *TGF- α* Transforming growth factor alpha, *VEGF* Vascular endothelial growth factor

## Conclusion

In the evolving landscape of liquid biopsy for HL, numerous crucial questions persist. The clinical integration of liquid biopsy demands thorough validation across diverse patient populations, necessitating exploration of optimal timing, frequency, and specific clinical contexts for its application. Integrative approaches spanning proteomics, genomics, and epigenomics hold the potential for innovative non-invasive tools, yet their exact contributions and clinical implications require further exploration. Robust validation through expanded patient cohorts is essential, considering potential variations in liquid biopsy performance across HL subtypes and stages. Moreover, understanding how liquid biopsy data can effectively inform treatment decisions and its economic implications compared to traditional imaging modalities is pivotal for its seamless integration into routine clinical practice, offering personalized strategies for managing this hematologic malignancy.

## Data Availability

Not applicable.

## Abbreviations

AuNPs: Gold nanoparticles
BEGEV: Bendamustine, gemcitabine and vinorelbine
cfDNA: Cell-free DNA
cHL: Classical Hodgkin Lymphoma
CNAs: Copy number alterations
ctDNA: Circulating tumor DNA
CyTOF: Mass cytometry
DEPArray: An image-based cell-sorting technology
DLBCL: Diffuse large B-cell lymphoma
EBV: Epstein-Barr Virus
EOT-PET: End-of-treatment PET
EVs: Extracellular vesicles
FDG-PET: 18-Fluoro-deoxyglucose positron emission tomography
FL: Follicular lymphoma
HL: Hodgkin Lymphoma
HRS: Hodgkin and Reed-Sternberg
iPET: Interim PET
LDCHL: Lymphocyte-depleted classical Hodgkin Lymphoma
LP: Lymphocyte-predominant
LRCHL: Lymphocyte-rich classical Hodgkin Lymphoma
MCCHL: Mixed cellularity classical Hodgkin Lymphoma
MHC-I: Major histocompatibility complex I
miRNAs: MicroRNAs
MRD: Minimal residual disease
NGS: Next-Generation Sequencing
NLPHL: Nodular lymphocyte-predominant Hodgkin lymphoma
NPV: Negative predictive value
NSCHL: Nodular sclerosis classical Hodgkin Lymphoma
PET/CT: Positron emission tomography/computed tomography
PET-0: Initial PET
PFS: Progression-free survival
PPV: Positive predictive value
scRNA-seq: Single-cell RNA sequencing
TARC: Thymus and activation-regulated chemokine
TME: Tumor microenvironment
VAFs: Variant allele frequencies
WES: Whole-exome sequencing
WGS: Whole-genome sequencing
